# Bureau of Gen. Sanitary Science, Etc.

**Published:** 1882-04

**Authors:** 


REPORT OF BUREAU OF GENERAL SANITARY SCIENCE, CLIMATOLOGY AND HYGIENE. To the American Institute, session 1881. Reprinted from "Transactions," By *B. W. James, M. D.*, Chairman. Jos. Eichbaum & Co., 48 Fifth Ave., Pittsburgh. 1881.

The report includes articles on the hygiene as applied to Medication, Dwellings, Occupation, Habits Formed, Fluids Drunk, Clothing Worn, Sewerage, with discussion. Thus it will be seen the report is both full and interesting, marred only by incompetent editing.

